# Assessment of biofeedback rehabilitation in post-stroke patients combining fMRI and gait analysis: a case study

**DOI:** 10.1186/1743-0003-11-53

**Published:** 2014-04-09

**Authors:** Silvia Del Din, Alessandra Bertoldo, Zimi Sawacha, Johanna Jonsdottir, Marco Rabuffetti, Claudio Cobelli, Maurizio Ferrarin

**Affiliations:** 1 Department of Information Engineering, University of Padova, Padova, Italy; 2 Neurology, Rehabilitation and Multiple Sclerosis Departments, IRCCS Don Gnocchi Found, Milano, Italy; 3 Biomedical Technology Department, IRCCS Don Gnocchi Found, Milano, Italy

**Keywords:** Stroke, fMRI, Gait, Biofeedback rehabilitation

## Abstract

**Background:**

The ability to walk independently is a primary goal for rehabilitation after stroke. Gait analysis provides a great amount of valuable information, while functional magnetic resonance imaging (fMRI) offers a powerful approach to define networks involved in motor control. The present study reports a new methodology based on both fMRI and gait analysis outcomes in order to investigate the ability of fMRI to reflect the phases of motor learning before/after electromyographic biofeedback treatment: the preliminary fMRI results of a post stroke subject’s brain activation, during passive and active ankle dorsal/plantarflexion, before and after biofeedback (BFB) rehabilitation are reported and their correlation with gait analysis data investigated.

**Methods:**

A control subject and a post-stroke patient with chronic hemiparesis were studied. Functional magnetic resonance images were acquired during a block-design protocol on both subjects while performing passive and active ankle dorsal/plantarflexion. fMRI and gait analysis were assessed on the patient before and after electromyographic biofeedback rehabilitation treatment during gait activities. Lower limb three-dimensional kinematics, kinetics and surface electromyography were evaluated. Correlation between fMRI and gait analysis categorical variables was assessed: agreement/disagreement was assigned to each variable if the value was in/outside the normative range (gait analysis), or for presence of normal/diffuse/no activation of motor area (fMRI).

**Results:**

Altered fMRI activity was found on the post-stroke patient before biofeedback rehabilitation with respect to the control one. Meanwhile the patient showed a diffuse, but more limited brain activation after treatment (less voxels). The post-stroke gait data showed a trend towards the normal range: speed, stride length, ankle power, and ankle positive work increased. Preliminary correlation analysis revealed that consistent changes were observed both for the fMRI data, and the gait analysis data after treatment (R > 0.89): this could be related to the possible effects BFB might have on the central as well as on the peripheral nervous system.

**Conclusions:**

Our findings showed that this methodology allows evaluation of the relationship between alterations in gait and brain activation of a post-stroke patient. Such methodology, if applied on a larger sample subjects, could provide information about the specific motor area involved in a rehabilitation treatment.

## Background

The ability to walk at the speeds and distances needed for home and community ambulation is an important and readily measured outcome after hemiplegic stroke. Six months after stroke, patients with persistent hemiparesis walk approximately one third as fast and only 40% the distance of age-matched healthy persons [1]. In this context additional gait training and rehabilitation treatments can improve walking speed and endurance [1]. The techniques based on biofeedback (BFB) have already been used extensively in various areas of rehabilitation, and several studies have used BFB, focusing on improving various aspects of gait in patients with chronic stroke, with encouraging results [2-6]. In normal gait, the ankle plantar flexors produce about 80% of the total energy necessary during the gait [7]. The work of the ankle plantar flexors is primarily used to contribute to the forward motion during gait, and thus it plays a fundamental role in determining gait velocity [5]. Patients with hemiparesis tend to have a severe reduction of ankle power in the push-off phase of gait as well as a much reduced velocity in gait.

Functional magnetic resonance imaging (fMRI), one of the main tools to investigate brain functional responses and follow up their evolution, has become a tool used to study recovery of hemiparesis [8], affecting 89% of stroke patients. Recently it has been investigated if fMRI could reflect training-related neural reorganization and provide a physiological insight into the optimal dose of additional gait training [9]. Walking is not feasible during fMRI. This is the reason why previous studies assessed cerebral activity while subjects imagine walking, but the cerebral resources used by an individual to visualize ambulation are open to wide variations across subjects [10]. Prior studies have used toe, foot, or knee movements during fMRI [1,8,9]. Such fMRI assessment, if applied serially over time, hopefully could provide information necessary to improve stroke recovery through development of rehabilitation strategies that are tailored to the individual [9]. Also gait analysis performed in laboratory settings has been shown to provide a great amount of valuable information, whose use for inferring the subject’s daily motor performance has already been assessed in the literature [11,12]. Focusing on walking, ankle dorsiflexion is of particular interest in patients with hemiparesis of the lower limb being an integral component in gait. It is not surprising that impairment of this movement is correlated strongly with walking difficulties and therefore predisposing these stroke patients to an increased likelihood of falling [13].

In this context, during fMRI, ankle dorsiflexion could be considered the most similar task to gait [1]. So far fMRI studies were based on the evaluation of cerebral activity during ankle dorsiflexion tasks to assay motor control during walking on healthy subjects [1,13]; moreover some studies assessed, with gait analysis techniques, the ability of task-oriented electromyography biofeedback rehabilitation to improve gait and gait associated neuroplasticity in post-stroke patients [11,12]. To the authors’ knowledge only one prior study combined motion capture with fMRI while subjects performed ankle dorsal-plantarflexion (ADPF) tasks [14]. Although the integrated system proposed by Casellato et al. 2010 showed different advantages with respect to state of art of fMRI, the motion capture system was only used to assess ankle kinematics while performing ADPF during fMRI without considering subject’ recovery after treatment in term of gait analysis outcome.

The aim of the present study was to integrate fMRI and gait analysis outcomes in order to investigate the ability of fMRI to reflect the phases of motor learning due to electromyographic biofeedback treatment during gait activities.

To evaluate potential relationships between activations in patients and their ability to walk, we used fMRI data to evaluate the cerebral activity during active and passive ADPF; afterward we investigated the correlations between the fMRI results and gait analysis data before and after 5 weeks of electromyographic biofeedback during gait rehabilitation. Therefore we present a pilot study to support the rationale for future use of the fMRI paradigm and its correlation with gait analysis outcomes for studies of the efficacy of rehabilitation treatments for walking. Such methodology, if applied over the time, will provide information about the specific motor area involved in a specific rehabilitation treatment in order to promote the development of rehabilitation strategies that are tailored to the individual.

## Methods

### Participants

A healthy control subject (CS) (45 years old, male, BMI 22, right-handed) was enrolled to evaluate the feasibility of different motor tasks as clinical protocols. One hemiparetic post-stroke subject (PS) was recruited. All subjects gave written informed consent. The protocol was approved by the local Ethics Committee (of the IRCSS Don Gnocchi, Milano). The patient (45 years old, male, BMI 25, right-handed, 6 years since stroke event) suffered from a chronic right hemiparesis due to hemorrhagic stroke, which caused a left lenticulocapsular lesion. The patient at the beginning of the study had a self-selected gait velocity of 0.68 m/sec and wore an orthosis during gait. He used a cane when walking outside. The patient was not claustrophobic and he had no implanted devices incompatible with fMRI. fMRI acquisitions were performed before (T1) and after (T2) electromyography (EMG) biofeedback rehabilitation. He underwent task oriented BFB consisting of a total of 15 rehabilitation sessions of 45 minutes, divided in three sessions every week [11,12].

### Training procedures

The biofeedback device was SATEM Mygotron (SATEM srl, Roma, Italy). EMG was band pass filtered between 20 and 950 Hz and amplified with a gain of 40,000 (50 μVrms range), then it was rectified and 100 ms averaged data were sampled at 150 Hz. A rehabilitation protocol was designed following the theorem of motor control learning [15]. The goal was to improve functional gait; thus, feedback was delivered during walk over ground. Electromyographic activity was recorded from the gastrocnemius lateralis and, under the hypothesis of being proportionally related to the push-off power, presented as an analogical audio signal to the patient; an auditory feedback tone was used to indicate whether push-off power met the target threshold. Target threshold was decided in the beginning of each session and was approximately 70% of maximum recruitment of gastrocnemius lateralis.

The therapeutic sessions were divided into phases with increasing variability of gait activities and decreasing application of feedback from first to last session. The aims of those phases were to improve gait performance, to increase patient’s auto error detection, and to transfer acquired skills during biofeedback condition to a context in which the feedback was no longer available [11,12]. The treatment phases were set up according to Jonsdottir et al., 2007 and 2010 [11,12].

### fMRI acquisition and data analysis

MRI data were collected with a 1.5 T Siemens Magnetom Avanto scanner (Erlangen, Germany).

Anatomic registration was acquired with a T1-weighted inversion recovery sequence with repetition time (TR) = 1900 ms; echo time (TE) = 3.37 ms; T1 = 1100 ms; flip angle = 15°; 176 slices - 1 mm thick; matrix 256 × 192; field of view (FOV) = 256 × 192 mm. For functional imaging sessions an echo-planar imaging (EPI) T2*-weighted sequence was used: TR =2500 ms, TE = 50 ms; matrix 64 × 64; FOV = 250 × 250 mm; voxel size = 3.9 × 3.9 × 5 mm; 25 axial slices. Subjects were asked to perform a blocked design consisting of 6 task–rest blocks. The subjects performed two different tasks: active (A) and passive (B) ankle dorsi/plantarflexion movements. The specific motor paradigm consisted of six 30 second periods of task B each one followed by 30 seconds of rest first for the left ankle and afterwards for the right ankle, and then six periods of task A (again followed by rest blocks) first for the left ankle and afterwards for the right (paretic) side, for a total of 4 trials. Each task-block start-stop event was based on acoustic signal. Each functional acquisition included 144 volumes.

1All data were first pre-processed using tools from the FMRIB Software Library (FSL, http://www.fmrib.ox.ac.uk/fsl) [16], applying the following procedures: motion correction, spatial smoothing using a Gaussian kernel of FWHM 8x8x8 mm. Following the pre-processing, the data were analyzed using MELODIC (Multivariate Exploratory Linear Optimised Decomposition into Independent Components), an implementation of probabilistic independent component analysis (PICA) [17], also part of FSL. Independent component analysis (ICA) is becoming a popular exploratory method for analyzing complex data such as those from fMRI experiments. On the basis of the PICA spatial/activation maps, where final maps were thresholded using an alternative hypothesis test based on fitting a Gaussian/Gamma mixture model to the distribution of voxel intensities within spatial maps and a posterior probability threshold of P > 0.5 threshold [17], a two-step process was used to identify the components of interest: first analyzing the peak of the power spectrum density (estimated by periodogram spectral estimation) together with the Z score of each component's time course. This frequency range of the peak was selected based on the power spectrum of the expected hemodynamic response function (HRF), which was in low-frequency range (<0.02 Hz). Next, the components’ spatial maps were visually inspected in the order determined by the previous step to identify the components with activation in the areas of interest and not areas that would not be related to the motor task.

Then the pre-processed fMRI images were used for a further data analysis which could be considered robust and standardized, thus allowing the identification of the quantitative indices which both described the neural activity and the enclosed clinical-physiological meaning.

An ad-hoc routine was implemented in MATLAB: unlike the previous statistical analysis, we analysed each of the voxels of the volume, but grouped into different Regions Of Interests (ROIs) by means of the Maximum Probability Hammersmith Brain Atlas [18].

In order to evaluate the quantitative indices, the probabilistic maps were coregistered: the entire brain volume was segmented with the help of a template into 116 anatomical ROIs (58 in the left hemisphere and 58 in the right one) [18]. Then the right and the left hemispheres were symmetrically divided so that each hemisphere was then partitioned into 58 corresponding ROIs. This partition was applied to all the functional images acquired. For each ROI the percentage of active voxels, with respect to the number of total voxels the ROI was made up, were evaluated. For each pair of corresponding ROIs (i.e. the i-th ROI of the right hemisphere, with the i-th ROI of the left hemisphere) the average differences between the percentage of active voxels of the contralateral (with respect to the moving limb) hemisphere ROI and the one of the ipsilateral hemisphere ROI were evaluated. This choice was made based on the evidence that the movement of a limb is controlled by the contralateral hemisphere. Using this value, a positive value of the average difference between the number of active voxels of the corresponding ROIs meant that the hemisphere contralateral to the movement was actually more active than the ipsilateral one, and conversely if the difference was negative. In order to describe and quantify the neural activity and the recovery after the rehabilitation treatment, an Index which represented differences between the number of active voxels of the corresponding ROIs was defined. The index (*diff Aft-Bef*), represented the change in the hemisphere differences detected before and after the rehabilitation treatment, so that a negative value meant that the difference of the percentage of active voxels between the two hemispheres got worse (decrease), conversely a positive value highlighted an improvement, this meant that after the rehabilitation treatment a greater interest of the contralateral side to the movement was observed, in fact, based on the literature, the contralateral hemisphere to the limb which was performing the task was expected to be more active than the ipsilateral hemisphere [1]. On the other hand if the ipsilateral hemisphere to the movement was recruited more than the other, it marked a “pathological” behaviour. Therefore, if therapy was not able to restore a “normal” condition, it could not be defined as a beneficial treatment as regards the neural activity.

All the fMRI indices were evaluated for four out of 58 brain areas which represented the ROI that should be the most involved during the execution of a motor task, for this study the motor area, the premotor area, the sensory-motor area and the cerebellum were considered.

### Motion capture system and gait data analysis

A 9 cameras motion capture SMART system (200 Hz, BTS, Milan, Italy), was used together with a dynamometric force plate (Kistler, Winterthur, Switzerland, 1000 Hz) in order to perform an instrumental gait analysis. EMG recordings of eight muscles (tibialis anterior, soleus, gastrocnemius lateralis, peroneus longus, rectus femoris, vastus lateralis, biceps femoris and hamstring) were made with an electromyographic 8-channel wireless device (Cometa S.r.l., Cisliano, Italy) at 1000 Hz. The three devices were synchronized. The LAMB protocol was adopted (total of 29 markers positioned on head, upper limbs, trunk, pelvis and lower limbs) in order to quantify the three-dimensional kinematics and kinetics of the subjects’ lower limbs [19-21]. Gait analysis was conducted on an 8 m walkway; three barefoot gaits either at self-selected (S-S) and fast (F) speed were collected.

The analysis focused on the most representative spatio-temporal and kinetic parameters, as described in [20], including Cadence, Stride Length, Ankle Power Peak, Ankle Positive Work, Ankle Negative Work, and Ankle Power Peak From Contralateral Heel Strike. Gait Speed (cm/s) was computed as stride length/stride duration and then normalized with respect to subject's height (%H/s). Cadence (stride/min) was computed as 60/stride duration. Kinetic parameters were computed from the time course of power produced/absorbed at the ankle joint during the stance phase. In particular, the peak of positive power was computed to quantify the produced power at the ankle at push-off (Ankle Power Peak), the positive positive/negative mechanical work (J/kg) was computed as the time integral of the produced/absorbed power. Moreover, in order to compute a motor control related parameter, the timing of the onset of the push-off positive power of the affected ankle was computed relative to the contralateral foot strike, and normalized to stride duration (% of stride duration): a positive (negative) value means that ankle power onset is after (before) the contralateral foot strike.

All parameters were considered for both the affected and the unaffected side, either for the walk at SS or at F speed and before (Bef) and after (Aft) BFB.

In total, for each acquisition, 28 parameters were considered (7 for each of the four combinations of speed and side).

The effectiveness of these parameters in capturing change in gait performance was demonstrated in previous studies [20,12]. Each patient’s gait parameter was compared with the corresponding speed-matching value of the normative bands obtained with the data of 20 normal subjects (mean age 43 ± 15, mean BMI 23 ± 5) [21]. This difference was evaluated as the absolute percentage difference (normalized by the normative value) with respect to the normative band. This allowed a comparison between the different gait parameters, in this way the parameters which differed more from the normative bands and those which were mostly influenced by the treatment could be highlighted.

For each one of the evaluated difference variables, an index was defined, in order to assess the effects of the EMG BFB treatment on the patients’ motor recovery, as the difference between the before (Bef) and after (Aft) BFB treatment values. The rehabilitation treatment, improving the motor performance, should reduce the evaluated difference: this was represented by a positive value of the *var Bef-Aft* index, thus because the patient data should be closer to the normative bands after the BFB rehabilitation treatment than before.

### Statistical analysis

Analysis of qualitative correlation was performed (*hetcor* function, Polycore Package, R Statistic software [22]) considering categorical variables. The qualitative correlation was based both on gait and fMRI categorical variables. The following categorical gait variables were considered: Speed, Cadence, Stride Length, Ankle Power Peak, Ankle Positive Work, Ankle Negative Work, and Ankle Power Peak From Contralateral Heel Strike in both conditions of normal and fast speed walking for both the tested walking condition (i.e. S-S and F walking speed). For each variable the normative bands (mean ± standard deviation) was evaluated by using the 20 normal control subjects’ data. Agreement or disagreement was assigned to each categorical variable respectively if the patient value was in the normative range (mean ± standard deviation) or outside the normative range. For the fMRI data agreement or disagreement were assigned for the following variables: normal activation of MI, diffuse activation of MI, no activation of MI for both active and passive ADP movements on both sides (see Table 1). The qualitative correlation was evaluated between the results before and after rehabilitation, first considering both gait and fMRI variables together, then only considering fMRI results before and after rehabilitation and finally only gait variables before and after rehabilitation.

**Table 1:** Example of the qualitative correlation table

**Categorical variables**					
**fMRI**	**Bef**	**Aft**	**Gait**	**Bef**	**Aft**
Ipsilateral task A			Ipsilateral		
M1 active	Agree	Agree	Speed in normative band	Disagree	Disagree
Diffuse activation of M1	Agree	Disagree	Cadence in normative band	Disagree	Disagree
M1 not active	Disagree	Disagree	Stride length	Agree	Agree
Contralateral task A			Ankle power peak in normative band	Disagree	Disagree
M1 active	Disagree	Disagree	Ankle positive work in normative band	Agree	Agree
Diffuse activation of M1	Disagree	Disagree	Ankle negative work in normative band	Disagree	Disagree
M1 not active	Disagree	Disagree	Ankle power peak from contralateral heel strike	Disagree	Agree
Ipsilateral task B			Contralateral		
M1 active	Disagree	Disagree	Speed in normative band	Disagree	Disagree
Diffuse activation of M1	Disagree	Disagree	Cadence in normative band	Disagree	Disagree
M1 not active	Disagree	Disagree	Stride length	Agree	Agree
Contralateral task B			Ankle power peak in normative band	Disagree	Disagree
M1 active	Disagree	Disagree	Ankle positive work in normative band	Agree	Agree
Diffuse activation of M1	Disagree	Disagree	Ankle negative work in normative band	Disagree	Disagree
M1 not active	Disagree	Disagree	Ankle power peak from contralateral heel strike	Agree	Agree

Example of table for evaluating qualitative correlation, the values were considered for both the self-selected and fast speed walking trials.

**Figure 1:**
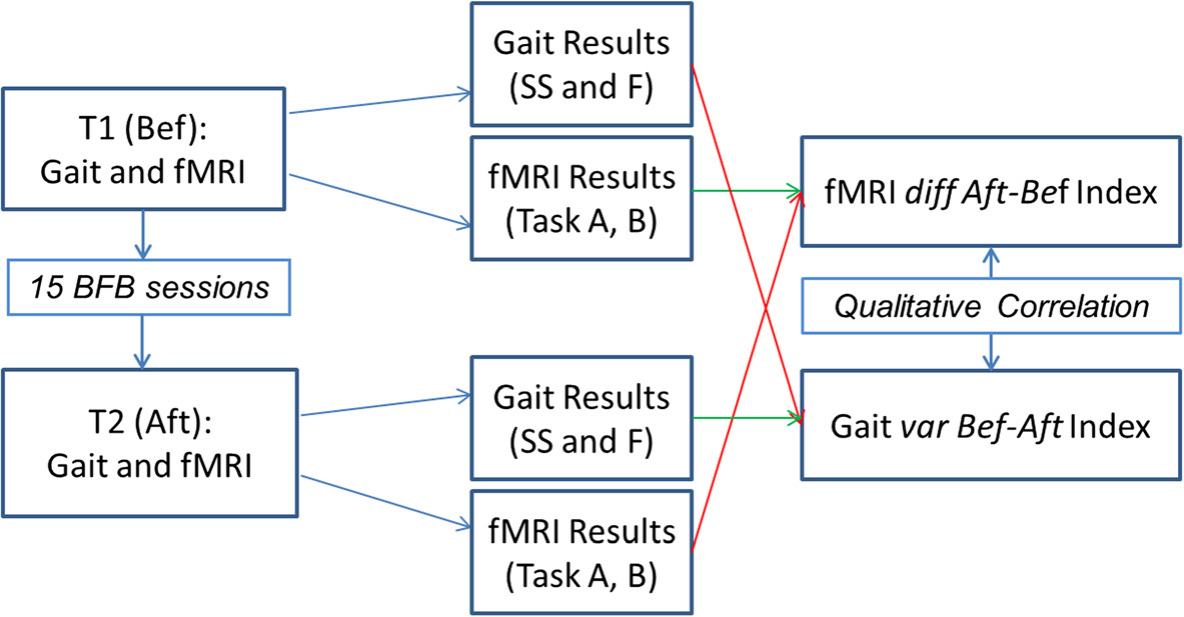
**Flowchart of the methodology.** Flowchart of the study and of the methodology: fMRI and GAIT Analysis were performed on the subject before (T0 (Bef)) and after (T1 (Aft)) the rehabilitation treatment (BFB). From each session gait analysis and fMRI results were evaluated, difference between the results of the gait analysis before and after the BFB treatment (*var Bef-Aft* Index) were evaluated, finally a quantitative correlation analysis between the gait and fMRI categorical variables was performed.

The methodology applied to data acquisition and analysis is summarized in Figure 1.

## Results

### FMRI results

**Figure 2:**
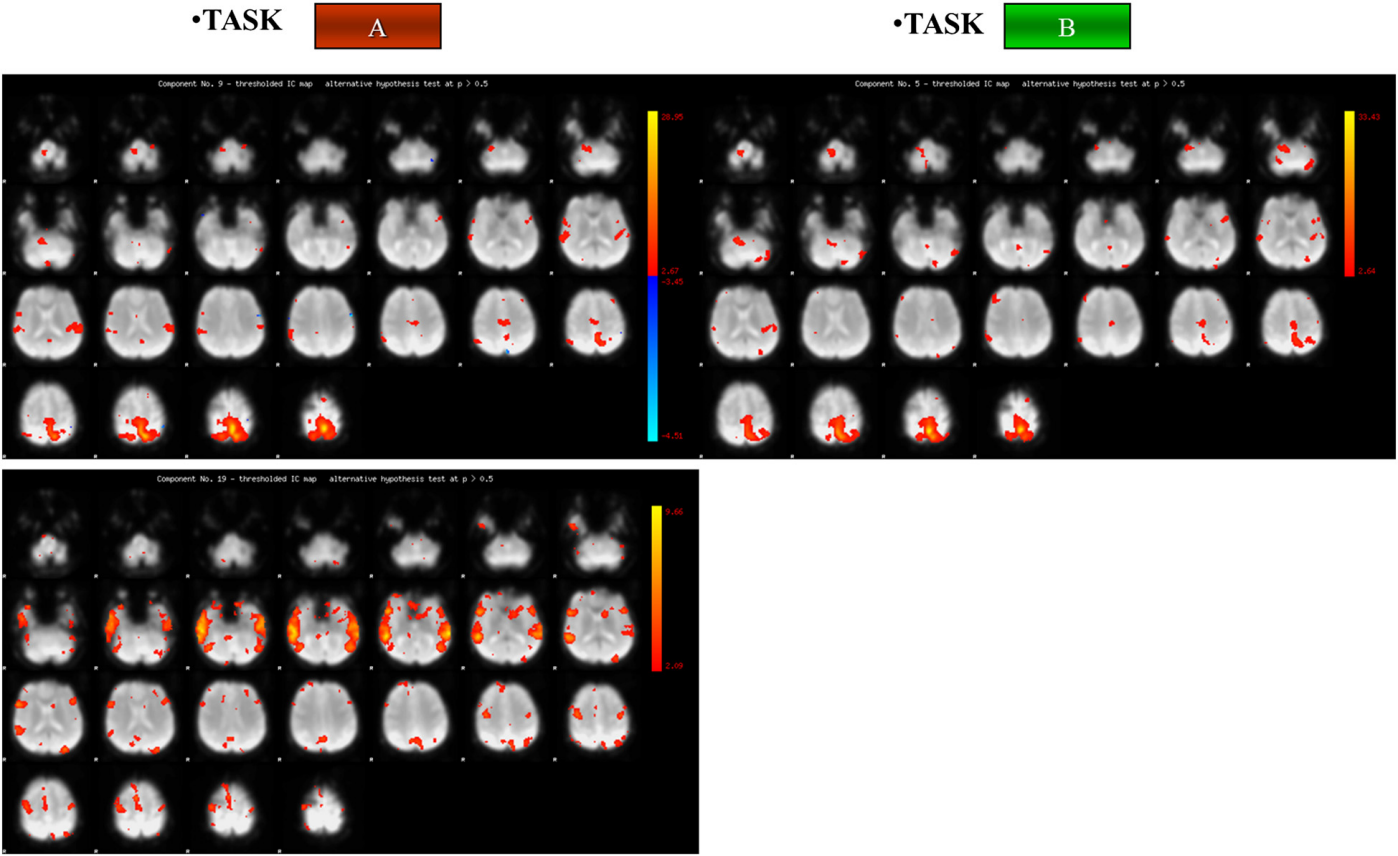
**Brain activation of the control subject.** Brain activation maps of the Control Subject for both task A (on the left) and task B (on the right). Brain activation maps of the Control Subject for both task A (on the left) and task B (on the right). The three scans are related to the components of interest identified by the PICA Analysis: in this figure on the right the two components identified for the active task (Task A) are shown, while on the left only a component was identified for the passive task (Task B). The Control Subject (right handed) performed Task A and Task B only with the right foot.

**Figure 3:**
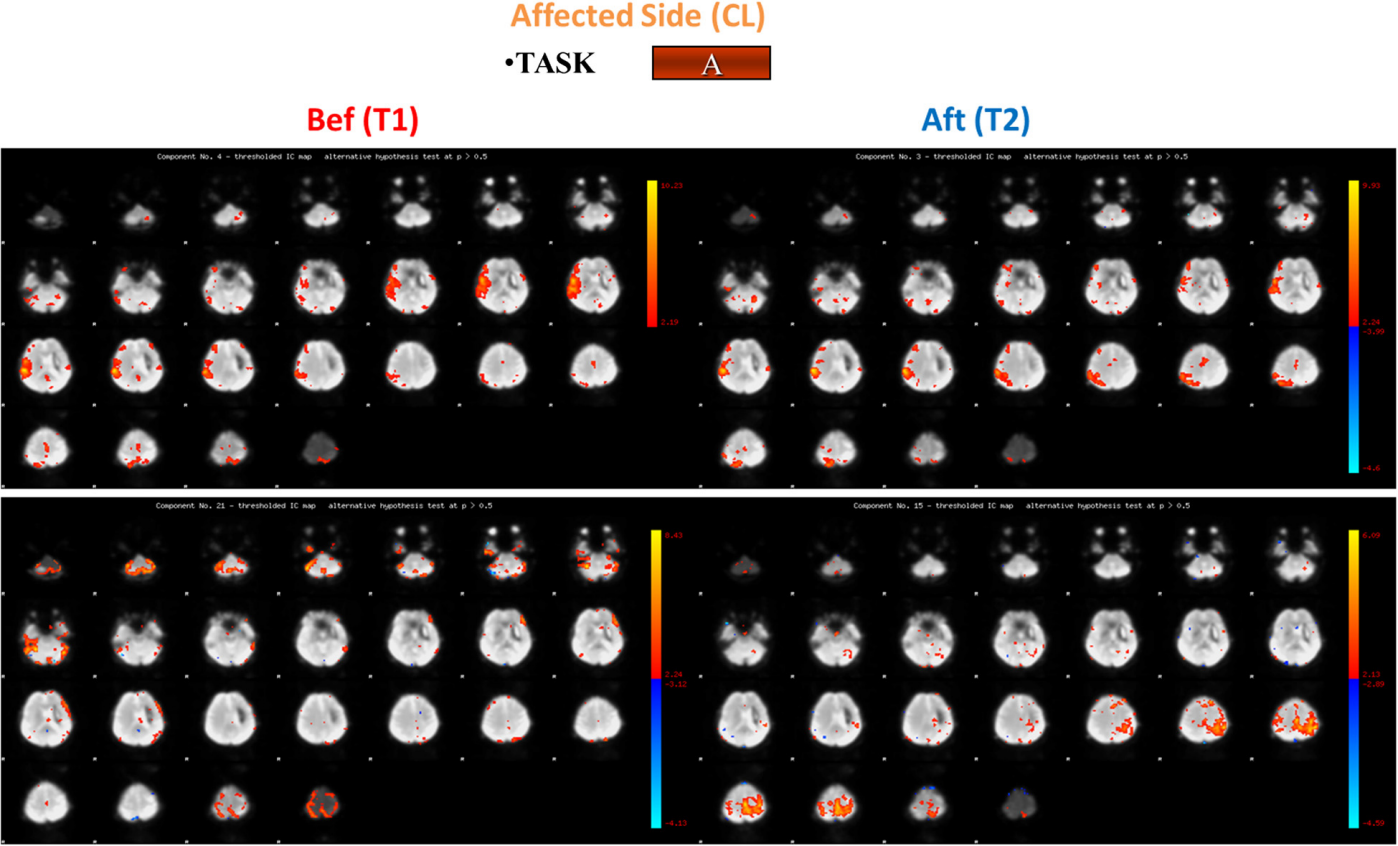
**Brain activation maps for the stroke patient: task A, affected side.** Brain activation for the Stroke Patient for task A for the affected side (contralateral (CL)) before (Bef) (on the left) and after (Aft) (on the right) the rehabilitation treatment. The four scans are related to the components of interest identified by the PICA Analysis: in this figure on the right the two components identified for the active task (Task A) before the rehabilitation treatment are shown, while on the left right the two components identified for the same task after the rehabilitation treatment are presented.

**Figure 4:**
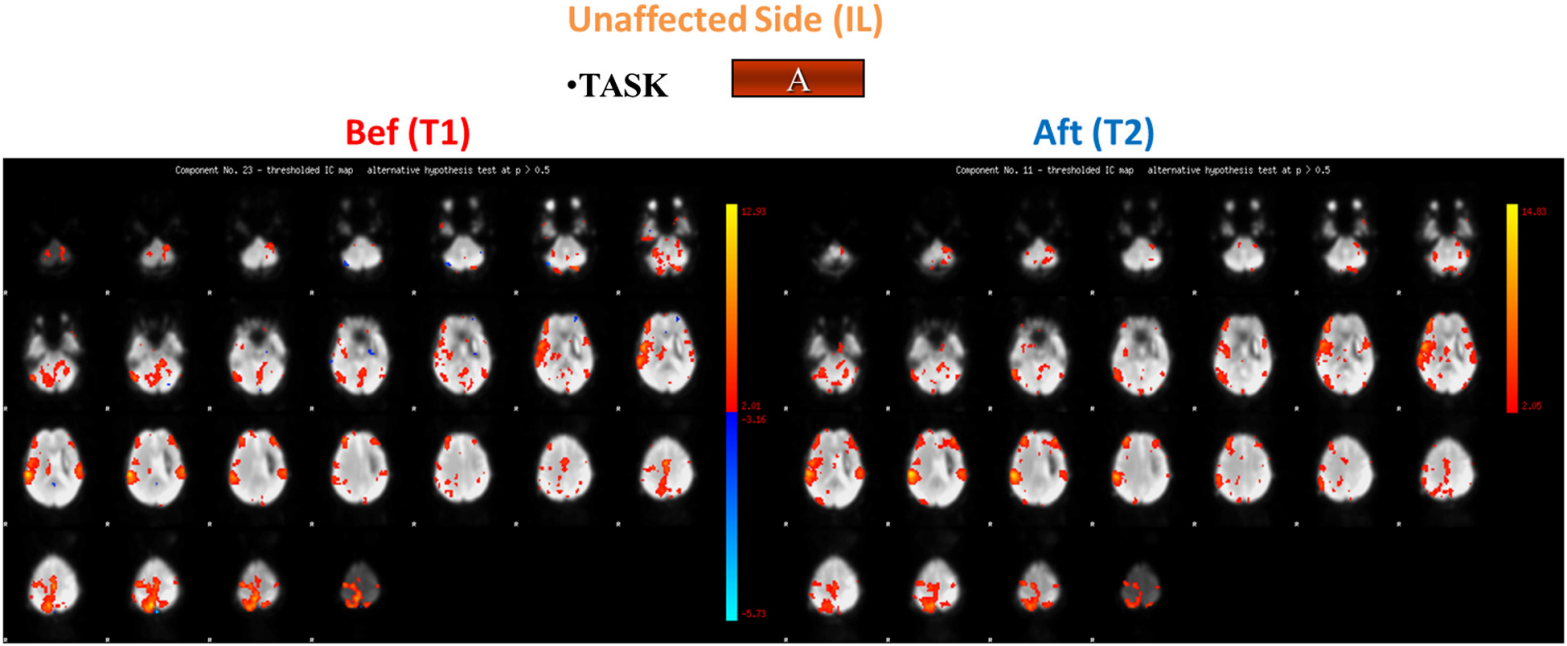
**Brain activation for the stroke patient: task A, unaffected side.** Brain activation for the Stroke Patient for task A for the unaffected side (ipsilateral (IL)) before (Bef) (on the left and after (Aft) (on the right) the rehabilitation treatment.

**Figure 5:**
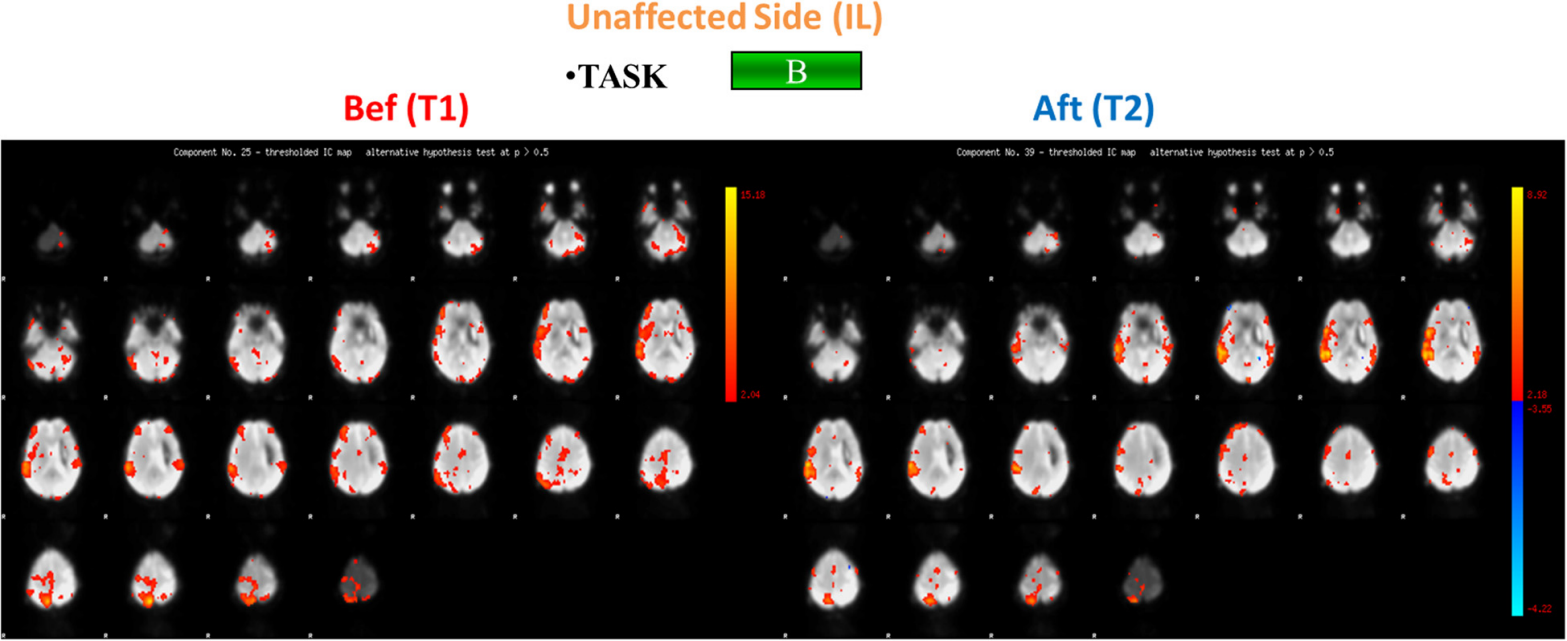
**Brain activation for the stroke patient: task B, unaffected side.** Brain activation for the Stroke Patient for task B (on the bottom) for the unaffected side (ipsilateral (IL)) before (Bef) (on the left) and after (Aft) (on the right) the rehabilitation treatment.

At time T1, concerning the active task A, individual brain activation maps revealed activation of the right primary motor area (MI) both for the control subject and for the patient when the left ankle was activated, in addition the premotor cortex was shown to be active for the patient (Figures 2 and 3); while considering the right paretic side of the patient a diffuse activation of the right pre-motor cortex and of the visual area was observed (Figure 3). For the passive task B the same brain activation of the task A were revealed for the control subject (Figures 1 and 2). Meanwhile when considering the patient’s data activation of the visual area was shown when the task was performed with the paretic side, a bilateral activation of the visual area, the activation of MI together with the cerebellum was revealed when the task was performed with the non paretic ankle (Figures 4 and 5).

**Figure 6:**
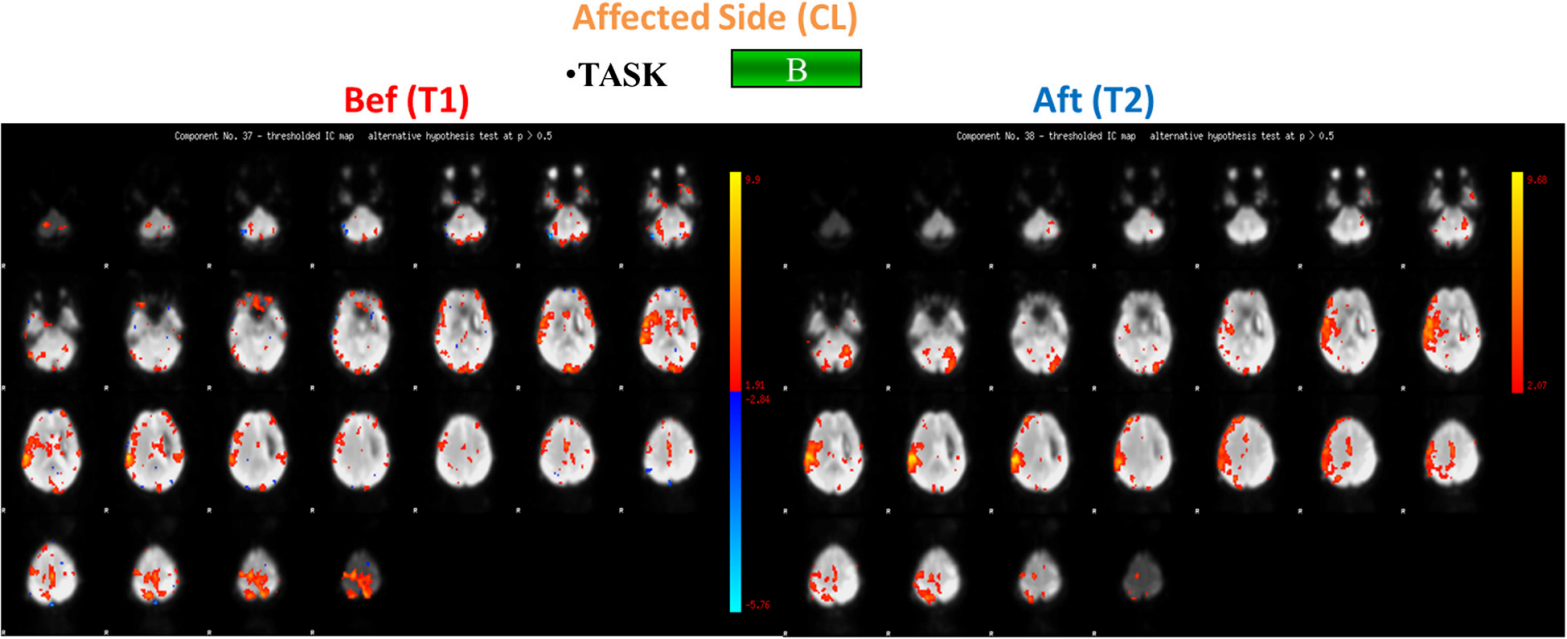
**Brain activation for the stroke patient: task B, affected side.** Brain activation for the Stroke Patient for task B for the affected side (contralateral (CL)) before (Bef) (on the left) and after (Aft) (on the right) the rehabilitation treatment.

After the rehabilitation treatment, at time T2, considering the affected side, the PS showed a confined activation of the right motor cortex for task A and a more limited activation of the visual area for task B (Figures 3 and 6). Besides, focusing on the unaffected side, we noticed the activation of MI, the premotor cortex together with the right motor cortex while the PS was performing the active task and as the visual area was shown to be active during Task B (Figures 4 and 5).

Results of the fMRI indices are shown in the Table 2. Considering the affected limb (i.e. the contralateral limb with respect to the brain lesion, in this case the right limb), the indexes and Figure 5 suggested an overall decreased, and more focused, activation for all the ROIs during task B, with both a greater interest of the contralateral brain areas and a reduced activation of the ipsilateral side with respect to the movement; while during task A the positive indexes suggest an overall improvement of the brain activity, more specifically, an increase in the activity of the left side of the motor area, the premotor area, and the sensory-motor area was observed, together with a significant decrease in the recruitment of the cerebellum activity (Figure 3). Different trends can be observed when considering the premotor and the sensory-motor areas: as regards task A, a positive index was found, suggesting an increased activity of the contralateral hemisphere, while concerning task B a negative value was found, this due to a decreased activation of the contralateral hemisphere and an increased (considering the premotor area) or stable (considering the sensory-motor area) activation of the ipsilateral hemisphere. No different trends were found considering the motor area and the cerebellum during the different tasks.

**Table 2:** **fMRI *diff Aft-Bef *** **index**

**Task**	**Task A**		**Task B**	
**Side**	**Contralateral**	**Ipsilateral**	**Contralateral**	**Ipsilateral**
Premotor area	7.29	-14.59	-13.44	-25.38
Sensory- motor area	35.56	11.01	-56.03	-5.95
Motor area	0.26	44.16	1.49	-13.69
Cerebellum	10.74	-21.69	17.60	19.87

fMRI *diff Aft-Bef Index* for the Post stroke patient for the contralateral and ipsilateral side, for both the active (A) and passive (B) task, values are expressed in percentage [%].

Considering the unaffected limb (i.e. the ipsilateral limb with respect to the brain lesion, in this case the left limb) before and after BFB an improved recruitment of the motor area was observed during task A, when an increased recruitment of the contralateral side was found. During both tasks the results showed a decreased activity of the pre-motor area. The recruitment of the cerebellum changed depending on the task: a decreased activation during task B and an increased activation during task A.

These results showed an overall modified neurological picture after the BFB treatment with a greater involvement of the affected hemisphere.

### Gait analysis results

**Figure 7:**
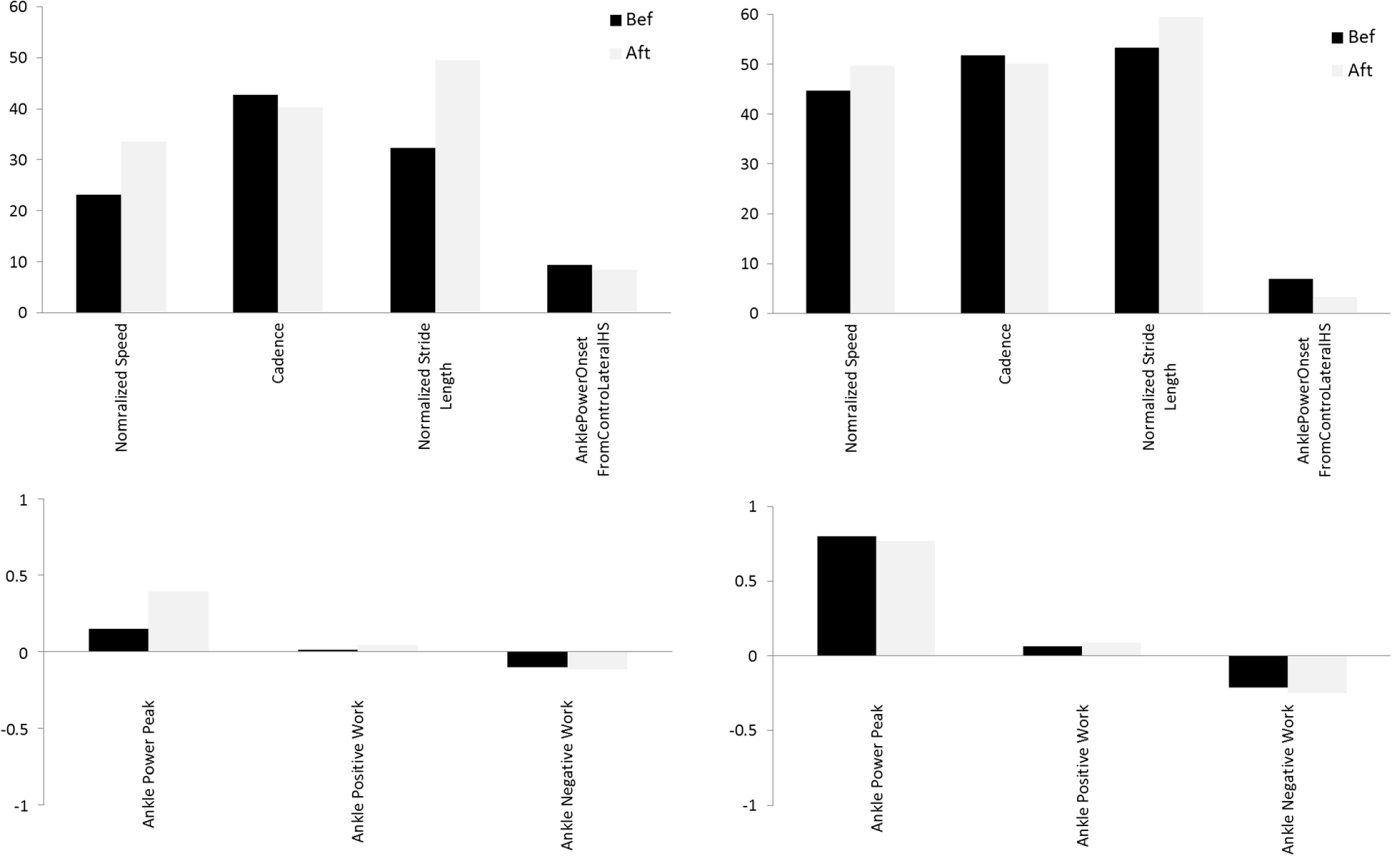
**Gait analysis results for the stroke patient.** Gait analysis results for the Stroke Patient: on the left the contralateral side parameters evaluated at self-selected speed, on the right the contralateral side parameters evaluated at fast speed. In black the before rehabilitation treatment results (Bef), in grey the after rehabilitation treatment results (Aft).

Comparing the PS gait data at time T1 and T2, cadence and stride length showed a trend towards the normal range, especially for self-selected gait velocity. Furthermore speed, ankle power, hip and ankle positive work increased. The graphics of the parameters evaluated both before and after BFB are shown in Figure 7. The PS showed increased cadence and Stride Length even though they are lower than the normative values, in addition, the ankle power peak greatly increased, the onset timing of the peak power with respect to the contralateral heel strike slightly improved even if an early onset was still found.

The values obtained for the *var Bef-Aft* index are reported in Table 3.

**Table 3:** **Gait *var Bef-Aft *** **index**

	**Bef-Aft percentage difference**	
**Gait index @ hemiplegic side**	**Self-aelected speed trials**	**Fast speed trials**
Speed	29.62	7.23
Cadence	-6.63	-3.19
StrideLength	31.18	8.22
AnklePowerPeak	17,63	-1.11
AnklePositiveWork	14.29	8.36
AnkleNegativeWork	8.70	32.26
AnklePowerOnsetFromControLateralHS	-82.42	-55.12

Gait *var Bef-Aft* Index for the Post stroke patient for the hemiplegic side, for both the walking at self-selected and fast speed, values are expressed in percentage [%].

Results revealed that most of all the considered parameters improved. More in detail, concerning the walking at SS speed, an increase of the stride length and of the speed together with a slight reduction of the cadence was observed. Furthermore both ankle power peak and ankle positive work greatly increased. As regards the walking at F speed, the stride length and the ankle positive work showed only a slight increase, while the ankle power peak resulted unchanged.

### Correlation results

The preliminary correlation results between fMRI and gait data showed a nice correlation (0.89 < R <0.99) between all the data for both the two sides together and for the affected side.

A good correlation (0.8 < R <0.99) for the fMRI data was found for both the contralateral and ipsilateral sides, while nice correlations coefficients were found for the gait data (R > 0.99), this could highlight that consistent changes occurred both in the brain activation patterns and in the gait.

## Discussion

The present study presents a new methodology based on both the fMRI and gait analysis outcomes, aiming at investigating a possible relationship between fMRI results of brain activation and changes in various gait parameters before and after task oriented biofeedback rehabilitation of a post stroke subject.

These preliminary results show that the stroke patient at time T1 before BFB rehabilitation exhibited altered cerebral activation with respect to the control subject, when requested to actively move the ankle of the paretic leg. These brain functional changes may represent a compensatory strategy designed to attempt in maintaining a normal performance despite scattered brain lesions. With this respect the indexes defined herein (differences between the number of active voxels of the corresponding ROIs) to describe and quantify the neural activity and the recovery after the rehabilitation treatment should also be considered an important contribution with respect to the state of art of fMRI data analysis. Indeed the evaluated fMRI index after the rehabilitation protocol evidenced a decreased, more focused activation of the brain areas during the execution of the movement with the affected limb (as seen also with the activation maps), with an increased involvement of the motor and premotor area with respect to the cerebellum. Considering the unaffected limb the motor area activation seemed to increase, especially during task A, while the premotor area decreased during both tasks, the cerebellum instead showed different trends during the two tasks (higher activation during task A, lower activation during task B). In general, after the rehabilitation treatment fMRI results showed a different neurological picture: the patient seemed to use both the affected and the unaffected hemisphere of the brain, with a greater and improved activation of the affected hemisphere; this was in agreement with gait analysis results that showed improvement in the motor outcome of the affected limb. It can be hypothesized that the functional improvement observed is reflected by the activation of the affected hemisphere. The increased activation of the motor area of the unaffected hemisphere indicates, however, general improvement in activation, possibly in response to a higher natural gait velocity following rehabilitation.

Gait analysis results showed that the BFB treatment could cause different outcomes: the PS seemed to have a more correct recovery of the motor patterns, the walking at SS speed greatly improved, meanwhile at F speed the parameters did not change accordingly. This could be due to the compensatory strategies developed by the patient during the F speed walking. Further, during rehabilitation more time was spent in gait activities at self-selected speed of the patient and this may have influenced the bigger effect observed on gait parameters during SS speed. As regards the evaluated gait index the patient showed improvements in both the space and time parameters, together with the ankle power peak and its timing of onset, that were associated to an increasing energy absorbed by the ankle.

The qualitative correlation analysis showed that from before to after the BFB treatment, the changes were consistent and related to both the fMRI data and to the gait results. The brain functional changes may be explained as compensatory strategy designed to achieve a more normal performance (assessed by gait analysis results) despite scattered brain lesions. Indeed an increased ankle power peak and more correct power peak onset timing were registered after BFB, thus suggesting that the overall motor performance improved.

The BFB treatment seemed to improve the neural activation patterns, showing a possible motor re-learning. With regard to the motor area, the activation pattern could suggest that the healthy part of M1 was active during the execution of the task of both the limbs, trying to balance the role previously carried out by the injured area.

From the observed values for the control subject and the patient, the motor area together with the cerebellum seemed to be the most relevant areas between those considered, being most involved during the execution of the voluntary movement. The significance of this index will need further study, first of all by increasing the number of subjects and by trying to evaluate its evolution over time.

An important limitation of the present study should be considered the limited sample of subjects, that shouldn’t be neglected when speculating on the clinical meaning of the results; however results can be considered encouraging in term of feasibility of the protocol.

Another important aspect that shouldn’t be neglected in the future fMRI acquisitions is the need to further immobilize the patient’s head in order to minimize images’ artefacts. During the early passive tasks the subjects sometimes tended to actively move or to resist, therefore subjects should be further trained in order to prevent such undesired voluntary muscular activation. Moreover, improvement could be made in order to help the patients to achieve a good timing during the active task. Finally, the evaluation of a normative band also for the fMRI could help to better interpret the different indices and the correlation values between the motor improvements and the outcome of the fMRI.

## Conclusions

This study aimed at evaluating the correlation between the gait analysis and the neural activity of a post-stroke patient before and after a rehabilitation treatment based on the use of electromyographic biofeedback during gait activities. Gait analysis and functional magnetic resonance imaging data were collected from the recruited subject, before and after the BFB treatment.

In order to describe both the brain activity and the motor behaviour in a quantitative and objective way, standard methods and innovative techniques of investigation were adopted.

Preliminary correlation analysis revealed that changes were observed for both the fMRI data, and the gait analysis data: this could be related to the possible effects BFB might have on the central as well as on the peripheral nervous system. Combining fMRI and gait analysis thus appears to be a valid approach to understanding changes that might occur in response to rehabilitation, in persons with hemiparesis.

These results, albeit preliminary, should be considered encouraging in terms of feasibility of the protocol and could be confirmed by incrementing the patients’ cohort. More appropriate statistical tests and analysis of quantitative correlation will be developed and refined once a larger sample of subjects will be available.

## Competing interests

Each of the authors has read and concurs with the content in the final manuscript. The contributing authors guarantee that this manuscript has not been submitted, nor published elsewhere. Each of the authors declares that don’t have any financial and non-financial competing interests.

## Authors’ contributions

Each of the authors has read and concurs with the content in the final manuscript. AB, ZS, JJ, MR, CC and MF participated in conceiving the study. SDD, AB, ZS, JJ, MR, CC and MF participated in its design and coordination and carried out the drafting of the manuscript. JJ and MR carried out the experimental part of the study relatives to the motion analysis and fMRI data collection and carried out and coordinated the gait data analysis. SDD participated to the experimental part of the study relatives to the fMRI data collection and performed the fMRI and correlation data analysis. All authors read and approved the final manuscript.
